# Sex differences in proximal femur shape: findings from a population-based study in adolescents

**DOI:** 10.1038/s41598-020-61653-4

**Published:** 2020-03-12

**Authors:** Monika Frysz, Jennifer Gregory, Richard M. Aspden, Lavinia Paternoster, Jonathan H. Tobias

**Affiliations:** 1 Musculoskeletal Research Unit, Translational Health Sciences, University of Bristol, Bristol, UK; 2 Arthritis and Musculoskeletal Medicine, University of Aberdeen, Aberdeen, UK; 3 Medical Research Council Integrative Epidemiology Unit, Bristol Medical School, University of Bristol, Bristol, UK; 4 Population Health Sciences, Bristol Medical School, University of Bristol, Bristol, UK

**Keywords:** Epidemiology, Bone

## Abstract

Hip shape is an important determinant of hip osteoarthritis (OA), which occurs more commonly in women. However, it remains unclear to what extent differences in OA prevalence are attributed to sex differences in hip shape. Here, we explore sex differences in proximal femur shape in a cohort of adolescents. Hip morphology was quantified using hip DXA scans from the Avon Longitudinal Study of Parents and Children. Independent modes of variation (hip shape mode (HSM) scores) were generated for each image using an adult reference statistical shape model (N = 19,379). Linear regression was used to examine sex differences for the top ten HSMs, adjusting for age, height, lean and fat mass. Complete outcome and covariate data were available for 4,428 and 4,369 participants at ages 14 and 18 years, respectively. Several HSMs showed sex differences at both time points. The combined effect of sex on hip shape at age 14 reflected flatter femoral head and smaller lesser trochanter in females compared with males and, following adjustment for age and body size, these differences became more pronounced. At age 18, smaller lesser trochanter and femoral neck width (FNW) in females still remained although differences in femoral head, femoral shaft and FNW were largely attenuated following adjustment. Sexual dimorphism in proximal femur shape can be discerned in adolescence and early adulthood. Observed differences in proximal femur shape, particularly at age 14 were largely independent of body size, however to what extent differences in hip shape in early life play a role in predisposing to hip OA in later life remains to be determined.

## Introduction

The overall incidence of osteoarthritis (OA) and, in particular, hip OA^1^ is greater in women compared with men, especially after the age of 50 years^2,3^. One explanation for this could be varying levels of hormones, in particular the rapid decrease of oestrogen around the time of menopause in women^4,5^. Another explanation for the differences in OA incidence might be differences in anatomy, including those present earlier in life. For example, one study, investigating joint space changes according to age and sex in asymptomatic hip joints (free from pain and radiological hip OA features), found that minimum hip joint space is smaller in women compared with men^6^. Alterations in hip morphology are thought to play an important role in the development of hip OA^6^, possibly via altered biomechanics, as demonstrated by higher peak stress in dysplastic hips compared with normal hips^7^. In addition, a previous finding that women have increased peak contact stress compared with men^8^ could partly explain higher incidence of hip OA in women.

With the majority of studies investigating the association between joint shape and OA in older adults and lack of longitudinal data with sufficient follow up, it is difficult to distinguish shape changes that are the direct result of OA from those that lead to OA development. While it is clear that joint shape can be altered by OA processes itself^9^, it has been recognized that abnormalities in hip morphology can also cause hip OA. On one hand developmental dysplasia of the hip is known to be associated with early-onset OA, while on the other hand subtle changes in hip structure and geometry are present in up to 90% of cases with primary hip OA^10^. However, little is known when these changes develop and how variation in hip morphology is shaped throughout life. Previous studies showed that cam-type deformity (known risk factor for hip OA^10–12^) arises in childhood^13^, often as a result of high impact activity^14^. To the extent that hip shape differences established in adolescence contribute to the risk of developing hip OA in later life, characterising hip shape changes around this time, including sex differences, is a prerequisite to understanding how early life factors influence the subsequent risk of hip OA. While the majority of studies investigating relationships with hip morphology relied on individual geometrical measures, given the limitations associated with these measures, Gregory *et al*.^15^ were the first to introduce statistical shape modelling (SSM) as a means of capturing shape of the proximal femur from images, using principal component analysis (PCA). SSM uses a set of landmark points to describe an outline of an object. This approach is used to study, describe and compare variations in anatomical shapes within and across different groups and has been previously applied to radiographs to investigate associations with incident radiographic hip OA (RHOA)^16^ and to predict total hip replacement (THR) in OA cases^17,18^.

A previous study of adolescents from the Avon Longitudinal Study of Parents and Children (ALSPAC) suggested that sexual dimorphism in hip geometry emerges around the time of puberty^19^. To determine whether differences in other aspects of proximal femur shape also emerge around this time, which may have relevance for the future risk of hip OA, we used the same cohort to characterize the systematic differences in proximal femur shape, as determined by SSM, between males and females.

## Methods

### Study participants

**Figure 1:**
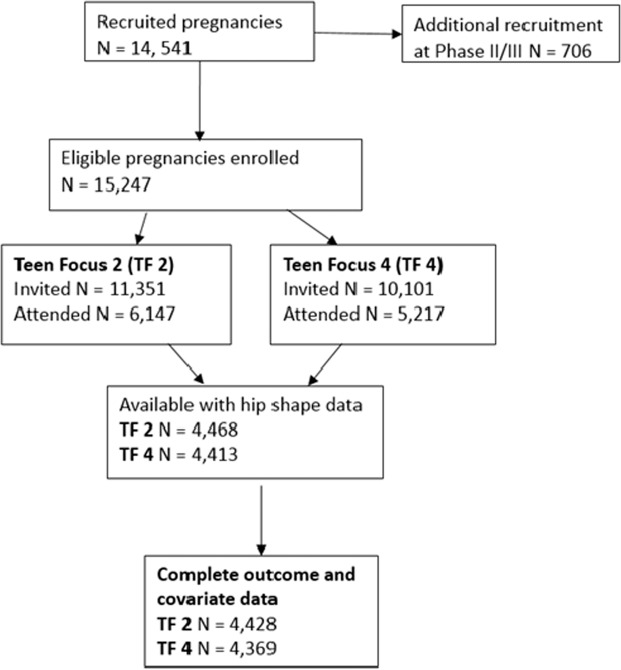
Flow diagram showing the distribution of ALSPAC participants from the recruitment to the present study population.

Pregnant women resident in Avon, UK with expected dates of delivery 1st April 1991 to 31st December 1992 were invited to take part in the study. The initial number of pregnancies enrolled is 14,541 (for these at least one questionnaire has been returned or a “Children in Focus” clinic had been attended by 19/07/99). Of these initial pregnancies, there was a total of 14,676 foetuses, resulting in 14,062 live births and 13,988 children who were alive at 1 year of age^20,21^. When the oldest children were approximately 7 years of age, an attempt was made to bolster the initial sample with eligible cases who had failed to join the study originally. As a result, when considering variables collected from the age of seven onwards (and potentially abstracted from obstetric notes) there are data available for more than the 14,541 pregnancies mentioned above. The total sample size for analyses using any data collected after the age of seven is therefore 15,247 pregnancies, resulting in 15,458 foetuses. Please note that the study website contains details of all the data that is available through a fully searchable data dictionary and variable search tool (http://www.bristol.ac.uk/alspac/researchers/our-data/). The present study is cross‐sectional, comprising general population sample acquired during follow‐up Teen Focus (TF) 2 and TF 4 research clinics. Prior to inclusion in the study, subjects were not screened for hip structural abnormalities. TF 2 was carried out between January 2005 and September 2006. Of 11,351 individuals invited 6,147 attended. TF 4 clinic started in December 2008 and was completed by mid-2011. Of 10,101 individuals invited, 5,217 attended. Figure 1 illustrates participant recruitment.

Ethical approval for the study was obtained from the ALSPAC Ethics and Law Committee and the Local Research Ethics Committees. TF 2 and TF 4 research clinics were approved by Central & South Bristol Research Ethics Committee (UBHT): 04/Q2006/168 and North Somerset & South Bristol Research Ethics Committee: 08/H0106/9, respectively. Informed consent for the use of data collected via questionnaires and clinics was obtained from participants and from parents and/or legal guardians following the recommendations of the ALSPAC Ethics and Law Committee at the time. Written parental and young person (YP) consent was obtained prior to performing DXA scan at TF 2 clinic. For YPs under 18 years of age, attending TF 4 clinic, prior to data collection consent was obtained from YP and a parent/guardian, if only YP was present verbal consent from parent/guardian was obtained over the phone and recorded on the consent form. Full details of the ALSPAC consent procedures are available on the study website (http://www.bristol.ac.uk/alspac/researchers/research-ethics/). All methods were performed in accordance with the relevant guidelines and regulations.

### Outcome and covariate data

Participant age at attendance, in completed months, was calculated as the difference between the date of attendance and date of birth, for both TF 2 and TF 4 clinics. Data on sex were obtained from hospital birth records. Total body (TB) lean and fat mass (kg) were measured using DXA scans, performed by GE Lunar Prodigy (Madison, WI, USA). Scans were acquired according to the manufacturer’s standard scanning and positioning protocols whereby the feet are internally rotated, and the positioning is fixed. Hip DXA scans acquired in the same way as TB scans were used to quantify the shape of proximal femur (outcome in this study). Height was assessed at the time of the DXA scan and was measured to the nearest 0.1 cm using a Harpender stadiometer with shoes removed.

### Statistical shape modelling

Statistical shape modelling was used to quantify the shape of the proximal femur from 2D hip DXA scans. Application of this method to ALSPAC data has been described previously^22^. Briefly, each hip DXA image was marked up with landmark points (key landmark points describing key anatomical features and remaining points evenly distributed between them) in Shape software (University of Aberdeen, UK) to build a 53-point statistical shape model (SSM). Following placement of landmark points, Procrustes analysis was used to estimate the mean shape. The aim of this step is first, to remove any translational, rotational and scaling information and then to align each image as closely as possible. After completing the alignment, PCA was performed to build the SSM. As previously reported^22^, reproducibility of point placement was assessed by Intraclass Correlation Coefficients (ICCs). The mean ICC values for the top ten HSMs were 0.87 for intra- and 0.70 for inter-observer agreement. In order to aid comparison with other cohorts and between the time points, a pre-defined set of points previously obtained from an adult reference population^23^ was applied to the ALSPAC offspring data. After PCA, independent modes of variation (hip shape modes (HSMs), were generated using the adult reference SSM. HSMs are ordered according to the amount of variance explained: the first mode accounts for the largest amount of variance in the dataset with subsequent modes accounting for less. Each individual is assigned a set of values for each HSM describing the distance from mean shape (in SDs).

### Statistical analysis

The normality of data was explored using descriptive statistics and histograms. Descriptive statistics are expressed as means with standard deviations (SD) for continuous variables. Linear regression was used to explore sex differences in the top ten HSMs (explaining 85% of variance) at each time point, and results are expressed as regression coefficients (β) indicating SD difference in the outcome (HSM scores) between males and females, 95% CIs and p value. The differences in mean HSM scores between males and females were explored in unadjusted analysis (model 1), and following adjustment for age at clinic attendance, height, lean and fat mass (model 2). All statistical analyses were performed using Stata version 14.0 (StataCorp, College Station, TX, USA).

To illustrate the overall sex differences in proximal femur shape at each time point, coefficients from linear regression from all modes showing evidence for sex differences (p < 0.005, based on Bonferroni-corrected P value cut-off; i.e. P = 0.05 divided by 10 to account for the 10 different modes being evaluated) were simultaneously entered into Shape software, to produce figures which represent the average overall male and female proximal femur shape. To aid visualisation the differences are magnified 4-fold. In addition, adjusted mean male and female shapes at age 14 and 18 years were overlaid in order to characterize the change in proximal femur shape between the time points.

## Results

### Population characteristics

Of 6,147 children who attended the TF 2 clinic, 4,428 had complete data on outcome and covariates. Their mean (SD) age was 13.8 (0.2) years. Boys were taller and had more lean mass compared with girls, whereas girls had greater fat mass compared with boys. Of 5,217 individuals who attended the TF 4 clinic, 4,369 had complete data on outcome and covariates. Mean (SD) age at clinic attendance was 17.8 (0.4) years and, similarly to TF 2, boys were taller and had more lean mass while girls had more fat mass compared with boys. Table 1 shows the characteristics of TF 2 and TF 4 participants.

**Table 1:** Characteristics of ALSPAC participants who attended TF 2 and TF 4 assessment clinics.

		Age 14			Age 18		
		N	Mean (SD)	p for sex diff*	N	Mean (SD)	p for sex diff*
Age (years)	Combined	4,428	13.8 (0.2)	0.36	4,369	17.8 (0.4)	0.59
	Male	2,117	13.8 (0.2)		1,931	17.8 (0.4)	
	Female	2,311	13.8 (0.2)		2,438	17.8 (0.4)	
Height (cm)	Combined	4,428	163.4 (7.6)	<0.001	4,369	171.2 (9.2)	<0.001
	Male	2,117	165.0 (8.7)		1,931	178.7 (6.6)	
	Female	2,311	162.0 (6.2)		2,438	165.2 (6.2)	
Fat mass (kg)	Combined	4,428	13.9 (8.0)	<0.001	4,369	18.4 (10.5)	<0.001
	Male	2,117	11.2 (7.7)		1,931	14.1 (10.0)	
	Female	2,311	16.4 (7.5)		2,438	21.8 (9.6)	
Lean mass (kg)	Combined	4,428	38.0 (6.4)	<0.001	4,369	45.7 (9.9)	<0.001
	Male	2,117	41.0 (7.2)		1,931	55.2 (6.1)	
	Female	2,311	35.2 (4.0)		2,438	38.1 (4.3)	

^*^Unpaired t-test to assess the null hypothesis of no difference in distributions between males and females at each time point.

### Sex differences in proximal femur shape at age 14

In unadjusted analysis, all modes, except HSM10, showed some evidence for sex differences (Table 2). Following adjustment for age, height, lean and fat mass associations with HSM4 attenuated slightly, HSM2 and HSM6 results were attenuated towards the null (and there was no longer evidence of an association), while several associations (HSM1, HSM3, HSM5, HSM7 and HSM8) were strengthened. In unadjusted analysis the difference between mean male and female HSM9 scores was 0.12 (positive coefficient indicating higher mean score in females). Following adjustment for age and body size (model 2), the difference in mean HSM9 score switched to negative (mean score in females now lower) (Table 2). Whilst positive HSM9 scores reflect smaller femoral head (inferior aspect proximal to lesser trochanter) and smaller lesser trochanter, negative HSM9 scores represent variation in the opposite direction i.e. both larger femoral head and lesser trochanter (please refer to ref. ^22^ for detailed description of the variation described by the top ten HSMs).

**Table 2:** Differences in hip shape mode scores between males and females (age 14).

HSM	Model 1		Model 2	
	β (95% CI)	p	β (95% CI)	p
1	0.13 (0.11,0.15)	<0.001	0.19 (0.16,0.22)	<0.001
2	0.06 (0.01,0.10)	0.013	−0.04 (−0.09,0.02)	0.163
3	−0.19 (−0.23,−0.15)	<0.001	−0.35 (−0.40,−0.30)	<0.001
4	0.22 (0.18,0.26)	<0.001	0.18 (0.13,0.23)	<0.001
5	−0.18 (−0.23,−0.14)	<0.001	−0.34 (−0.39,−0.28)	<0.001
6	−0.14 (−0.18,−0.10)	<0.001	−0.03 (−0.08,0.02)	0.210
7	0.18 (0.15,0.22)	<0.001	0.23 (0.19,0.28)	<0.001
8	−0.73 (−0.78,−0.67)	<0.001	−0.87 (−0.93,−0.80)	<0.001
9	0.12 (0.08,0.17)	<0.001	−0.08 (−0.14,−0.03)	0.004
10	−0.01 (−0.04,0.03)	0.737	−0.02 (−0.06,0.03)	0.478

Abbreviations: HSM (hip shape mode), CI (confidence interval). Table shows linear regression coefficients (β) indicating SD difference in the outcome (HSM scores) between males and females (N = 4,428), 95% CIs and p value. Positive beta coefficients indicate higher mean HSM scores in females, compared with males. Model 1: unadjusted, model 2: adjusted for age, height, lean and fat mass.

**Figure 2:**
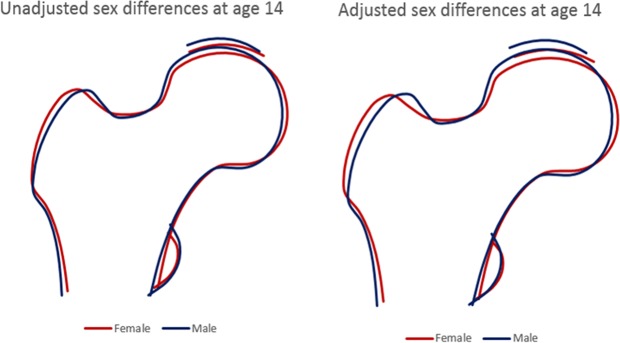
Sex differences in hip shape at age 14 based on the difference in mean HSM scores. Sex beta coefficients (for all modes with p < 0.005; model 1: HSM1, HSM3–9, model 2: HSM1, HSM3–5, HSM7–9) magnified 4-fold were modelled in Shape to represent the average overall hip shape between males and females.

The overall effect of sex on proximal femur shape at age 14, after combining sex differences across different HSMs, is shown in Fig. 2. In the unadjusted model, differences in femoral head shape were observed between sexes, with the femoral head appearing flatter in females. In addition, in females, the lesser trochanter was smaller, and the greater trochanter was positioned more laterally. After adjusting for age and body size, if anything, sex differences in femoral head and greater trochanter shape became more pronounced, while those in lesser trochanter size became less marked.

### Sex differences in proximal femur shape at age 18

In unadjusted analysis, there was strong evidence for sex differences in all modes except HSM1 and HSM7 (Table 3). Following adjustment for age, height, lean and fat mass (model 2) associations of sex with HSM2, HSM5, HSM6 and HSM9 were partially attenuated, while the association with HSM3, HSM4, HSM8 and HSM10 was strengthened.

**Table 3:** Differences in hip shape mode scores between males and females (age 18).

HSM	Model 1		Model 2	
	β (95% CI)	p	β (95% CI)	p
1	0.02 (−0.003,0.05)	0.084	0.09 (0.03,0.14)	0.001
2	0.48 (0.43,0.52)	<0.001	0.41 (0.31,0.51)	<0.001
3	0.13 (0.09,0.17)	<0.001	0.17 (0.09,0.25)	<0.001
4	0.15 (0.11,0.20)	<0.001	0.19 (0.10,0.28)	<0.001
5	0.39 (0.34,0.44)	<0.001	0.29 (0.19,0.39)	<0.001
6	−0.60 (−0.65,−0.55)	<0.001	−0.53 (−0.63,−0.43)	<0.001
7	0.003 (−0.04,0.04)	0.872	−0.09 (−0.18,−0.01)	0.036
8	−0.25 (−0.31,−0.20)	<0.001	−0.31 (−0.42,−0.20)	<0.001
9	0.49 (0.43,0.54)	<0.001	0.33 (0.22,0.44)	<0.001
10	−0.07 (−0.11,−0.02)	0.004	−0.16 (−0.25,−0.06)	0.001

Abbreviations: HSM (hip shape mode), CI (confidence interval). Table shows linear regression coefficients (β) indicating SD difference in the outcome (HSM scores) between males and females (N = 4,369), 95% CIs and p value. Positive beta coefficients indicate higher mean HSM scores in females, compared with males. Model 1: unadjusted, model 2: adjusted for age, height, lean and fat mass.

**Figure 3:**
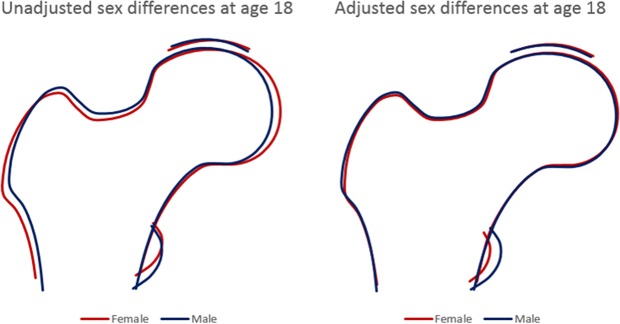
Sex differences in hip shape at age 18 based on the difference in mean HSM scores. Sex beta coefficients (for all modes with p < 0.005; model 1: HSM2–6, HSM8–10, model 2: HSM1–6, HSM8–10) magnified 4-fold were modelled in Shape to represent the average overall hip shape between males and females.

The overall effect of sex on proximal femur shape at age 18 is shown in Fig. 3. In the unadjusted model, differences were broadly similar to those observed at age 14. In females, the femoral head appeared flatter and to extend more medially, the lesser trochanter was smaller, and the greater trochanter extended more laterally. In addition, females had a narrower femoral neck width (FNW), and wider femoral shaft. Following adjustment for age and body size, these differences were considerably attenuated, although a smaller lesser trochanter and slightly narrower FNW in females was still observed.

### Differences in proximal femur shape between age 14 and 18 years

**Figure 4:**
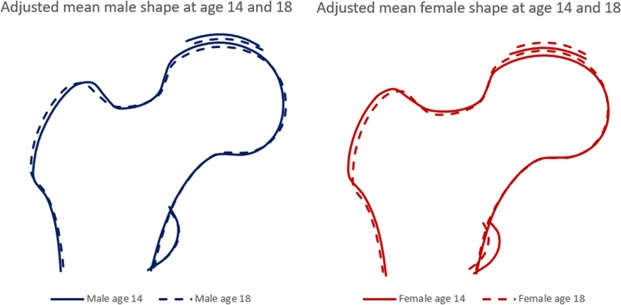
Mean male and female proximal femur shapes at age 14 and 18 years.

Figure 4 shows mean proximal femur shapes at age 14 and 18 years, stratified by sex. In both sexes, the femoral head was relatively flatter at age 14 compared with age 18. In addition, in females, the lesser trochanter was smaller at age 14 compared with age 18.

## Discussion

We have presented the first, comprehensive description of sex differences in proximal femur shape in adolescents, as assessed by SSM performed on hip DXA scans, in a cohort of ALSPAC offspring at ages 14 and 18 years. Substantial sex differences were observed, ranging from −0.73 to 0.22 SDs, and from −0.60 to 0.49 SDs at the age of 14 and 18 years, respectively (based on unadjusted analyses). On combining results across multiple modes, at age 14, differences were observed in shape of the femoral head and greater trochanter, and in size of the lesser trochanter. Broadly similar differences were observed at age 18, with the addition that females also showed a narrower FNW but a wider femoral shaft. Following additional adjustment for sex differences in body size and composition, the shape differences observed at age 14 were, if anything, magnified whereas those at 18 were largely attenuated, except for differences in lesser trochanter size which remained, as did small differences in FNW.

The differing effects of adjustment at age 14 and 18 suggest that distinct pathways underlie sex differences in proximal femur shape at these two ages. Results for adjusted analyses suggested that sex differences in body size and composition are largely responsible for those in proximal femur shape at age 18. In contrast, sex differences at age 14, which were not directly related to differences in body size and composition, may reflect those in skeletal maturity. In line with this suggestion, at the time of age 14 DXA scans, ALSPAC females were a mean of two years past age of peak height velocity (PHV), in contrast to males who were on average at PHV^24^. Furthermore, at age 14, differences in greater trochanter and femoral head shape were seen between early versus late maturing males^25^, which are reminiscent of sex differences in proximal femur shape at age 14 reported here. Taken together, these findings support the suggestion that differences in skeletal maturation make an important contribution to observed sex differences in proximal femur shape at age 14.

When looking at mean male and female proximal femur shapes at both time points, the most marked changes were observed in superolateral aspect of the femoral head, whereby between the age of 14 and 18 years the femoral head became flatter in males compared with females. Taken together, we found that the femoral head takes on a flatter appearance during adolescence, and the fact that girls are more skeletally mature at age 14 compared to boys likely contributes to more marked sex differences at age 14. To our knowledge, this study is the first time SSM has been applied to describe variation in proximal femur shape in a cohort of adolescents and young adults. One previous cross-sectional study in adults from the Medical Research Council National Survey of Health and Development (NSHD) (mean age 63 years), which also used SSM, reported sex differences in proximal femur shape in older adults^26^. Consistent with our findings at age 18, men in the NSHD cohort had greater FNW compared with women. The authors also observed flattening of the femoral head in women which we also observed in females at age 14 in the present study, but not in the adjusted results at age 18. Flattening of femoral head has been found to be a risk factor for hip OA in previous studies. For example, using SSM to quantify femoral head and neck on standard pelvic radiographs, Gregory *et al*. reported that the development of hip OA was associated with change in mode 1 scores which represented flattening of the femoral head^17^. Similarly, in a study of elderly women, higher mode 5 scores (reflecting relatively flatter femoral head in the superior aspect compared with more curved medial aspect of the femoral head) predicted incident RHOA^27^.

Whilst there are no studies exploring sex differences in global shape of the proximal femur in adolescents, previous studies explored sex differences in other aspects of hip morphology. For example, using landmark-based geometrical measures to radiographs of the lower half of the body (including aspects of femoral head, greater and lesser trochanters and distal femur), Pujol *et al*. reported sex differences in the shape of the femur from age 11 upwards in Spanish males and females between the ages of 9–16 years, and 9–14 years, respectively^28^. In particular, the authors reported that females had smaller lesser trochanters compared with males, a finding consistent with our results. However, whereas Pujol *et al*. also reported smaller greater trochanters in females, we found sex differences in shape as opposed to size per se. A number of studies have investigated sex differences in measures associated with femoroacetabular impingement (FAI). For example, in a study of 132 patients (mean age, 15 years; range, 12–18 years; 45 male, 87 female) which used CT scans to define femoral morphology, the mean alpha-angle was greater in males compared with females whilst no differences in the femoral head-neck offset was found between the sexes^29^. Another study in a group of 2,081 healthy participants (874 male, 1207 female; mean age, 18.6 years) reported that radiographic features suggestive of FAI, such as pistol-grip deformity and flattening of the lateral femoral head are more common in males compared with females^30^. Whilst these results cannot be directly compared with the results of this study, it is clear that sex differences in femoral morphology can be discerned in adolescence and future studies are justified to investigate relationships between measures of global proximal femur shape with measures of FAI.

The shape parameters that we found to differ according to sex in adolescents and young adults, have shown some previous evidence for association with risk of hip OA. Reduced femoral head sphericity in older adults was previously found to predict risk of total hip replacement up to ten years from baseline^31^ and was also found to be associated with hip OA in cases with symptomatic RHOA compared with asymptomatic controls with no signs of RHOA^11^. Since the upper femoral head had a somewhat flatter appearance in females compared to males (the difference being most pronounced at age 14), theoretically, this might contribute to the greater risk of hip OA observed in females compared with males. In addition, to the extent that the smaller lesser trochanter size in females may be artefactual due to greater internal rotation, this finding might reflect a tendency towards joint hypermobility which could also represent a contributory factor in hip OA. However, to what extent the variation in lesser trochanter size and position represents anatomical variation as opposed to subject positioning during image acquisition is currently unknown.

### Strengths and limitations

One of the strengths of this study includes the use of SSM, an approach to describing overall shape of the proximal femur, which has been applied in a large cohort of adolescents and young adults to describe variation in proximal femur shape. Whilst SSM describes the global shape of the proximal femur, one of the drawbacks of this method is the fact that it is difficult to determine which particular aspects of proximal femur shape may be related to the development of a disease. Since the present study used 2D images, the changes we observed could have reflected factors such as rotation and positioning, rather than true differences in hip shape. This contrasts with SSM applied to 3D images such as those derived from hip CT scans^32^. Whereas the impact of positioning and rotation is minimised during hip DXA scans by standardized operating procedures, this is unable to account for variation in 3D position within the scanner as a result of differences in distribution of body tissue. Approximately 98% of cohort participants were of white European descent. Given the previously reported ethnic differences in hip morphology^33^ these results are likely to be generalisable to European populations only. Whilst we have shown that there are sex differences in proximal femur shape during adolescence and link this with evidence from previous studies of OA to hypothesise that such changes might to some extent underpin the sex differences observed in OA in later life, further work is required to determine any causal relationship. In addition, whilst structural abnormalities in hip shape are a well-recognized risk factor for hip OA, calculations based on pelvic anatomy suggested that this could be explained by estimates of higher contact stresses in the hip joint^7,34,35^. To what extent hip contact stress depends on subtle variation in joint shape and, in early life, differs between males and females is unknown. Whether this could underpin the sex differences observed in OA in later life remains to be established. Whilst it would be interesting to estimate hip joint force and stress distribution for participants in our study, we were unable to do so given the fact that standard anteroposterior radiographs were not collected.

## Conclusions

We used SSM applied to hip DXA scans to investigate sex differences in proximal femur shape in a large cohort of adolescents and young adults. At age 14, compared to males, females had a somewhat flatter femoral head, smaller lesser trochanter and laterally positioned greater trochanter. At age 18 in unadjusted analysis, similar differences were observed to those seen at age 14, however these were largely attenuated following body size adjustment, whilst some differences in FNW and femoral shaft persisted. Sex differences in proximal femur shape at age 14 were reminiscent of those previously observed in relation to puberty, suggesting they reflect sex differences in skeletal maturation. In contrast, sex differences in body size and composition appeared to be largely responsible for those in proximal femur shape observed at age 18. Further studies are justified to determine whether sex differences in proximal femur shape we observed, particularly those at age 18 which are expected to persist, contribute to sex differences in the risk of hip OA in later life.

## Data Availability

ALSPAC data access is through a system of managed open access. The steps below highlight how to apply for access to the data 1. Please read the ALSPAC access policy (PDF, 627 kB) which describes the process of accessing the data and samples in detail, and outlines the costs associated with doing so. 2. You may also find it useful to browse our fully searchable research proposals database, which lists all research projects that have been approved. 3. Please submit your research proposal for consideration by the ALSPAC Executive Committee using the online process. You will receive a response within 10 working days to advise you whether your proposal has been approved. If you have any questions about accessing data, please email: alspac-data@bristol.ac.uk.
